# Supplementary material for: Structure-preserving visualization for single-cell RNA-Seq profiles using deep manifold transformation with batch-correction
Source: Commun Biol. 2023 Apr 4;6:369. doi: 10.1038/s42003-023-04662-z (PMC10073100; doi:10.1038/s42003-023-04662-z)
Supplement: Supplementary file 2 — Supplementary Information [file 42003_2023_4662_MOESM2_ESM.pdf]

Structure-Preserving Visualization for Single-cell RNA-Seq Profiles Using Deep Manifold Transformation with Batch-Correction – Supplementary Information

Yongjie Xu[1,2], Zelin Zang[1,2], Jun Xia[1,2], Cheng Tan[1,2], Yulan Geng[2], and Stan Z. Li[2,*]

[1]Zhejiang University, Hangzhou, 310058, China
[2]AI Division, School of Engineering, Westlake University, Hangzhou, 310024, China
[*]Corresponding author e-mail: stan.zq.li@westlake.edu.cn

Supplementary Note 1: Global/hierarchical and local structure preservation criteria

Following Poin_maps[1], to quantitatively compare the performance of different embedding methods, we use scale-independent quality criteria proposed by Lee and Verleysen[2]. The main idea is that a good dimensionality reduction method will have good preservation of local and global distances on the manifold, e.g., close neighbors should be placed close to each other while maintaining large distances between distant points. Below we provide a short summary of how to compute this metric. All the details can be found in the original paper of Poin_maps[1] and Lee et al.[2].

DV receives a scRNA-seq dataset $\mathscr{D} = \left\{(\mathbf{x_i}, \mathbf{y_i})_{i=1}^{N}\right\}$ as input, where $\mathbf{x_i} \in \mathbb{R}^d$ is the gene expression vector of cell i, $\mathbf{y_i}$ is a categorical variable vector specifying the batch in which $\mathbf{x_i}$ is measured, d is the number of measured genes, and N is the number of cells. Let $\mathbf{X^h} = \left\{\mathbf{x_1^h}, ..., \mathbf{x_N^h}\right\}$ denote the high-dimensional data matrix and d_{ij}^h denote the distance from $\mathbf{x_i^h}$ to $\mathbf{x_j^h}$ in the high-dimensional space, and $\mathbf{X^l} = \left\{\mathbf{x_1^l}, ..., \mathbf{x_N^l}\right\}$ denote the low-dimensional embedding matrix and d_{ij}^l denote the distance from $\mathbf{x_i^l}$ to $\mathbf{x_j^l}$ in the low-dimensional space. Assume $d_{ij}^h = d_{ji}^h$ and $d_{ij}^l = d_{ji}^l$, the distances could be used to compute high ρ_{ij}^h and low ρ_{ij}^l dimensional ranks between the points:

$$\rho_{ij}^h = \mid \left\{k : d_{ik}^h < d_{ij}^h \text{ or } \left(d_{ik}^h = d_{ij}^h \text{ and } 1 \leq k < j \leq N\right)\right\} \mid \tag{1}$$

$$\rho_{ij}^l = \mid \left\{k : d_{ik}^l < d_{ij}^l \text{ or } \left(d_{ik}^l = d_{ij}^l \text{ and } 1 \leq k < j \leq N\right)\right\} \mid \tag{2}$$

where $\mid A \mid$ denotes the cardinality of set A. According to this definition, reflexive ranks are set to zero ($\rho_{ii}^h = \rho_{ii}^l = 0$) and ranks are unique, i.e. there are no ex aequo ranks ($\rho_{ij} \neq \rho_{ik}$ for $k \neq j$). This means that non-reflexive ranks belong to $\{1, \ldots, N-1\}$.

A co-ranking matrix $\mathbf{Q} = [q_{rs}]_{1 \leq r,s \leq N-1}$ can be defined as:

$$q_{rs} = \mid \left\{(i,j) : \rho_{ij}^h = r \text{ and } \rho_{ij}^l = s\right\} \mid \tag{3}$$

where Q is the joint histogram of the ranks and is actually a sum of N permutation matrices of size $N-1$. It contains all the necessary information about how ranks are preserved in a given low-dimensional representation.

As was demonstrated by Lee and Verleysen[2], the co-ranking matrix $\mathbf{Q}$ is straightforward to compute, and it could be used to compute Q_{NX}-scale-independent quality criteria for dimensionality reduction for a given value of $K = 1, \ldots, N-1$.

$$Q_{NX}(K) = \frac{1}{KN} \sum_{(r,s) \in \mathbb{UL}_K} q_{rs} \tag{4}$$

where $\mathbb{UL}_K = \{1, \ldots, K\} \times \{1, \ldots, K\}$ is the upper left corner of co-ranking matrix. $Q_{NX}(K) \in [0,1]$ assesses the overall quality of the embedding. Essentially, it measures the preservation of K-ary neighborhoods. A perfect embedding has $Q_{NX}(K) = 1$ for every $K = 1, \ldots, N-1$.

The left part of the Q_{NX} curve reflects how local properties are preserved, and the right part corresponds to the preservation of global properties. To improve its readability, Lee et al. proposed to use two scalar quality criteria Q_{local} and Q_{global} focusing separately on low and high dimensional qualities of the embedding:

$$Q_{\text{local}} = \frac{1}{K_{\max}} \sum_{K=1}^{K_{\max}} Q_{NX}(K) \tag{5}$$

$$Q_{\text{global}} = \frac{1}{N - K_{\max}} \sum_{K=K_{\max}}^{N-1} Q_{NX}(K) \tag{6}$$

where K_{max} defines the split of the $Q_{NX}(K)$ curve and is automatically computed as:

$$K_{\max} = \arg\max_K \left(Q_{NX}(K) - \frac{K}{N-1}\right) \tag{7}$$

The quantities of Q_{local} and Q_{global} range from 0 (bad) to 1 (good).

In this work, we adopt Euclidean distance to estimate distances d_{ij}^h in the high-dimensional space (input data). For Poincaré maps, we use geodesic distances estimated as the length of a shortest-path in a k-nearest neighbors graph according to the original tutorial. For the distances d_{ij}^l in the low-dimensional space, we use Euclidean distances for all the embeddings except DV_Poin, DV_Lor and Poincaré maps, for which we use hyperbolic distances. For large datasets, 5000 samples are randomly subsampled for evaluation.

Supplementary Note 2: Benchmarks on datasets

The cord blood mononuclear cell dataset[3] consists of 8617 cells, including 8009 cord blood mononuclear cells and 608 mouse 3T3 fibroblasts, produced by the CITE-seq protocol[4] on the 10× Chromium (v2) platform[5]. We adopt the preprocessed data in scPhere[6], which including CD14+ erythroid cells and the first ten erythrocytes in the dataset, and they selected 2000 highly variable genes based on the Seurat5 tutorial[7].

Human splenic NK cells are from a study profiling human and mouse splenic, and blood NK cells[8], and profiles by 10× Chromium (v2). We use the 1755 human splenic NK cells from donor one and adopt the preprocessed data in scPhere[6], which selected 2724 highly variable genes, partitioned the 1755 cells into four groups, and labeled them as hNK_Sp1, hNK_Sp2, hNK_Sp3, hNK_Sp4.

Human lung cells are from asthma patients and healthy controls[9], and profiles by either 10× Chromium or Drop-seq[10]. We use the preprocessed 3314 cells in scPhere[6] from a donor prepared by the Drop-seq protocol.

The mouse white adipose tissue stromal cell dataset contains 1378 cells from mouse white adipose tissue[11] profiled by 10× Chromium (v2). In the original study, the authors only analyzed 1045 tdTomato- mGFP+ cells and identified adipocyte precursor cells (APC), fibro-inflammatory progenitors (FIP), committed preadipocytes, and mesothelial cells. We adopt the preprocessed data in scPhere[6], which analyzed all the cells and further identified pericytes, macrophages, and two groups of doublets.

The reginal ganglion cell atlas (RGC) dataset consists of 35,699 mouse retinal ganglion cells profiled by 10× Chromium (v2)[12]. The original analysis identified 45 clusters, and one cluster consisted of two cell types. We adopt the preprocessed data in scPhere[6], which selected 2724 highly variable genes.

We use 599,926 human cell landscape cells (HCL)[13] from human fetal or adult tissues profiles by the Microwell-Seq platform[14]. In the original study, these cells were portioned into 102 clusters, and 77 of 102-cell clusters can be grouped into six major cell groups: fetal stromal cells, fetal epithelial cells, adult endothelial cells, endothelial cells, adult stromal cells and immune cells. We adopt the preprocessed data in scPhere[6], which selected 2724 highly variable genes. Specially, For heterogeneous case in "batch invariant" model verification experiment, we input the raw data due to the demand of whole gene names. We reclassify the training set and test set according to tissue name to satisfy complex multilevel batch factors. The 43 tissues in training set include 'AdultAdrenalGland_3', 'AdultBladder_2', 'AdultEsophagus_2', 'AdultGallbladder_1', 'AdultHeart_2', 'AdultKidney_2', 'AdultKidney_3', 'AdultLiver_2', 'AdultLiver_4', 'AdultLung_1', 'AdultLung_3', 'AdultOmentum_1', 'AdultOmentum_2', 'AdultPeripheralBlood_2', 'AdultPeripheralBlood_4', 'AdultStomach_2', 'AdultStomach_3', 'AdultThyroid_2', 'BoneMarrow_2', 'CordBloodCD34P_2', 'CordBlood_2', 'FetalAdrenalGland_2', 'FetalAdrenalGland_3', 'FetalBrain_3', 'FetalBrain_4', 'FetalBrain_5', 'FetalHeart_1', 'FetalIntestine_1', 'FetalIntestine_4', 'FetalIntestine_5', 'FetalIntetsine_3', 'FetalKidney_3', 'FetalKidney_4', 'FetalKidney_5', 'FetalLung_2', 'FetalMaleGonad_2', 'FetalPancreas_1', 'FetalPancreas_2', 'FetalRib_3', 'FetalSkin_2', 'FetalStomach_2', 'FetalThymus_1' and 'Liver_1', and the 28 tissues in test set include 'AdultAdrenalGland_2', 'AdultBladder_1', 'AdultEsophagus_1', 'AdultGallBladder_2', 'AdultHeart_1', 'AdultKidney_4', 'AdultLiver_1', 'AdultLung_2', 'AdultOmentum_3', 'AdultPeripheralBlood_3', 'AdultStomach_1', 'AdultThyroid_1', 'BoneMarrow_1', 'CordBloodCD34P_1', 'CordBlood_1', 'FetalAdrenalGland_4', 'FetalBrain_6', 'FetalHeart_2', 'FetalIntestine_2', 'FetalKidney_6', 'FetalLung_1', 'FetalMaleGonad_1', 'FetalPancreas_3', 'FetalRib_2', 'FetalSkin_3', 'FetalStomach_1', 'FetalThymus_2' and 'Liver_2'. In addition, the celltypes of confusion matrix in Supplementary Fig. 9c are arranged in 'M2 Macrophage', 'AT2 cell', 'Hepatocyte/Endodermal cell', 'Gastric chief cell', 'Proliferating T cell', 'Antigen presenting cell (RPS high)', 'Fetal Neuron', 'Fetal fibroblast', 'Fetal stromal cell', 'Intermediated cell', 'Ventricle cardiomyocyte', 'Intercalated cell', 'Proximal tubule progenitor', 'Sinusoidal endothelial cell', 'Fetal epithelial progenitor', 'Fetal enterocyte ', 'Loop of Henle', 'Mast cell', 'T cell', 'Fetal endocrine cell', 'Fibroblast', 'Smooth muscle cell', 'Fetal chondrocyte', 'Monocyte', 'Dendritic cell', 'Fetal neuron', 'B cell', 'Epithelial cell (intermediated)', 'Stratified epithelial cell', 'Thyroid follicular cell', 'Ureteric bud cell', 'Erythroid progenitor cell (RP high)', 'Neutrophil (RPS high)', 'Neutrophil', 'Macrophage', 'Fetal acinar cell', 'Epithelial cell', 'Fetal mesenchymal progenitor', 'B cell (Plasmocyte)', 'Stromal cell', 'Endothelial cell (APC)', 'Goblet cell', 'Endothelial cell (endothelial to mesenchymal transition)', 'Primordial germ cell', 'Fetal skeletal muscle cell', 'Erythroid cell', 'CB CD34+', 'Fasciculata cell', 'Basal cell' and 'Endothelial cell'.

We use mouse cell atlas cells (MCA)[13] profiles by the Microwell-Seq platform for heterogeneous cases in the "batch invariant" model verification experiment when training data and testing data belong to different species. We remove the batch gene background and perform analysis using the same pipeline and parameters as for the HCL described by the original tutorial.

The cells of the colon mucosa are from 68 biopsies collected from 18 ulcerative colitis patients and 12 healthy individuals[15], and profiles by 10× Chromium (either v1 or v2). We adopt the prepocessed data in scPhere[6], which obtain a total of 301,749 cells (26,678 stromal cells and glia, 64,457 epithelial cells, and 210,614 immune cells as annotated in the original study) by filtering likely low-quality cells (clusters). The cells span 12 stromal cell types/states, 12 epithelial cell types/states, and 23 immune cell types/states, identified by unsupervised clustering and manual annotations. They used Seurat to select 1307, 1361, and 1068 highly variable genes for the three major cell types, respectively. The dataset is profiled in a complex experimental

design from the colon mucosa of 18 patients with ulcerative colitis (UC), a major type of inflammatory bowel disease (IBD), and 12 healthy individuals. In addition to each individual patient biopsy being a batch, there are many other factors to consider in the original study: individuals were either healthy or with UC, cells were collected separately from the epithelial and lamina propria fractions of each biopsy, and there were two replicate biopsies for each healthy individual and as a pair of inflamed and uninflamed biopsies for the UC patients (for a few UC patients, there were replicate inflamed and/or replicate uninflamed biopsies). Finally, samples were collected at two time periods, separated by over a year (analyzed as train and test data in the original study).

The C. elegans embryonic cell dataset consists of 86,024 cells[16] profiled using 10× Chromium (v2). The embryo times are partitioned into 12-time bins, and 63.5% of the cells are assigned to 36 major cell types based on annotation from GEO: GSE126954. We adopt the preprocessed data in scPhere[6] and treat the cells with embryo time in the range 100–130 as the root cells because embryo time <100 consisted mostly of germline cells that are also observed in embryo time >650.

Supplementary Note 3: Supplementary tables

Supplementary Tab. 1, Supplementary Tab. 2 and Supplementary Tab. 3 describe the hyperparameters used by DV_Eu, DV_Poin and DV_Lor on the experimental datasets, respectively. Moreover, Supplementary Fig. 11 verifies the robustness of DV_Eu to hyperparameter on processing large *static* scRNA-seq data with/without batch effect problem. Supplementary Fig. 12 verifies the robustness of DV_Poin and DV_Lor to hyperparameter on processing *dynamic* scRNA-seq data with batch effect problem.

Supplementary Table 1. Hyperparameters of DV_Eu in experimental datasets.

	Datasets	Hyperparameters				
		learning rate	batch size	ν^{vi}	γ	β
Single-batch	CD14+ monocytes	0.0005	100	0.005	10	
	Human lung cells	0.0001	500	0.001	100000	0
	Human splenic NK cells	0.0005	100	0.001	1000	0
	Mouse white adipose tissue stromal cells	0.0005	100	0.005	10	0
	Reginal ganglion cell atlas	0.001	500	0.001	100000	0
	Human cell landscape cells	0.0005	2000	0.01	1000	0
	C. elegans embryonic cells	0.0005	500	0.001	10	0
Multi-batch	Colon mucosa cells	0.0005	2000	0.01	10	1
	Stromal cells	0.005	2000	0.05	100000	1
	Epithelial cells	0.001	2000	0.05	10	100
	Immune cells	0.001	500	0.001	100000	100
	C. elegans embryonic cells	0.005	3000	0.01	10	0.01
Pre-trained DV	Stromal cells	0.005	2000	0.01	100000	1
	Epithelial cells	0.005	500	0.005	10	10000
	Immune cells	0.001	500	0.01	10	1
	Human cell landscape cells	0.0005	2000	0.005	1000	0

∗ The annotations of hyperparameters can be found in *choices of hyperparameters* subsection in *Methods* section.

Supplementary Table 2. Hyperparameters of DV_Poin in experimental datasets.

	Datasets	Hyperparameters				
		learning rate	batch size	ν^{vi}	γ	β
Single-batch	CD14+ monocytes	0.0005	500	0.01	10	0
	Human lung cells	0.005	100	0.05	1000	0
	Human splenic NK cells	0.005	100	0.001	10	0
	Mouse white adipose tissue stromal cells	0.005	100	0.005	10	0
	Reginal ganglion cell atlas	0.005	500	0.001	10	0
	Human cell landscape cells	0.005	1000	0.01	1000	0
	C. elegans embryonic cells	0.005	3000	0.001	100000	1000
Multi-batch	Epithelial cells	0.001	500	0.005	10	100
	C. elegans embryonic cells	0.0005	2000	0.001	10	10000

Supplementary Table 3. Hyperparameters of DV_Lor in experimental datasets.

	Datasets	Hyperparameters				
		learning rate	batch size	v^{vi}	γ	β
Single-batch	CD14+ monocytes	0.0005	500	0.005	10	0
	Human lung cells	0.005	100	0.05	1000	0
	Human splenic NK cells	0.01	100	0.001	10	0
	Mouse white adipose tissue stromal cells	0.001	100	0.005	10	0
	Reginal ganglion cell atlas	0.005	500	0.001	10	0
	Human cell landscape cells	0.001	1000	0.01	1000	0
	C. elegans embryonic cells	0.001	500	0.001	10	0
Multi-batch	Epithelial cells	0.001	500	0.005	10	100
	C. elegans embryonic cells	0.005	3000	0.001	1000	0.01

Supplementary Note 4: Supplementary figures

Supplementary Fig. 1 and Supplementary Fig. 2 note the results of DV on visualizing small scRNA-seq datasets. Supplementary Fig. 3 notes the results of DV on visualizing large *static* scRNA-seq datasets labeled by cell type. Supplementary Fig. 4 notes the results of DV on addressing complex technical and biological batch for visualization and analysis in colon biopsies from healthy individuals and UC stromal patients, and Supplementary Fig. 6 notes the results from healthy individuals, UC stromal, epithelial and immune patients. Supplementary Fig. 7 and Supplementary Fig. 8 note the results of DV_Eu on using complex batch vectors to pinpoint cell types impacted by biological factors and to generate a batch-invariant reference atlas on UC stromal and epithelial datasets, respectively. Supplementary Fig. 9 generates a pre-trained reference atlas and maps heterogeneous scRNA-seq data. Supplementary Fig. 10 notes the convergence speed rapidly on large *static* and *dynamic* scRNA-seq data without batch effect problem. Supplementary Fig. 11 verifies the robustness of DV_Eu to hyperparameter on processing large *static* scRNA-seq data with/without batch effect problem. Supplementary Fig. 12 verifies the robustness of DV_Poin and DV_Lor to hyperparameter on processing *dynamic* scRNA-seq data with batch effect problem. Supplementary Fig. 13 notes the Poincaré disk embedding of DV_Poin, which highlights the progression of C. elegans embryonic cells in time without batch effect problem. Supplementary Fig. 14 and Supplementary Fig. 15 note the Poincaré disk embeddings of DV_Lor and scPhere_wn respectively, which highlight the progression of C. elegans embryonic cells in time with batch effect problem. Supplementary Fig. 16 gives the runtime of all methods when visualizing single-cell datasets (with batch effect problem) with different cell numbers. Supplementary Fig. 17 makes the ablation study of DV when visualizing single cell datasets with batch effect problem.

References

1. Klimovskaia, A., Lopez-Paz, D., Bottou, L. & Nickel, M. Poincaré maps for analyzing complex hierarchies in single-cell data. *Nat. communications* **11**, 1–9 (2020).
2. Lee, J. A. & Verleysen, M. Scale-independent quality criteria for dimensionality reduction. *Pattern Recognit. Lett.* **31**, 2248–2257 (2010).
3. Amodio, M. *et al.* Exploring single-cell data with deep multitasking neural networks. *Nat. methods* **16**, 1139–1145 (2019).
4. Stoeckius, M. *et al.* Simultaneous epitope and transcriptome measurement in single cells. *Nat. methods* **14**, 865–868 (2017).
5. Zheng, G. X. *et al.* Massively parallel digital transcriptional profiling of single cells. *Nat. communications* **8**, 1–12 (2017).
6. Ding, J. & Regev, A. Deep generative model embedding of single-cell rna-seq profiles on hyperspheres and hyperbolic spaces. *Nat. communications* **12**, 1–17 (2021).
7. Butler, A., Hoffman, P., Smibert, P., Papalexi, E. & Satija, R. Integrating single-cell transcriptomic data across different conditions, technologies, and species. *Nat. biotechnology* **36**, 411–420 (2018).
8. Crinier, A. *et al.* High-dimensional single-cell analysis identifies organ-specific signatures and conserved nk cell subsets in humans and mice. *Immunity* **49**, 971–986 (2018).
9. Vieira Braga, F. A. *et al.* A cellular census of human lungs identifies novel cell states in health and in asthma. *Nat. medicine* **25**, 1153–1163 (2019).
10. Macosko, E. Z. *et al.* Highly parallel genome-wide expression profiling of individual cells using nanoliter droplets. *Cell* **161**, 1202–1214 (2015).

11. Hepler, C. *et al.* Identification of functionally distinct fibro-inflammatory and adipogenic stromal subpopulations in visceral adipose tissue of adult mice. *Elife* **7**, e39636 (2018).

12. Tran, N. M. *et al.* Single-cell profiles of retinal ganglion cells differing in resilience to injury reveal neuroprotective genes. *Neuron* **104**, 1039–1055 (2019).

13. Han, X. *et al.* Construction of a human cell landscape at single-cell level. *Nature* **581**, 303–309 (2020).

14. Han, X. *et al.* Mapping the mouse cell atlas by microwell-seq. *Cell* **172**, 1091–1107 (2018).

15. Smillie, C. S. *et al.* Intra-and inter-cellular rewiring of the human colon during ulcerative colitis. *Cell* **178**, 714–730 (2019).

16. Packer, J. S. *et al.* A lineage-resolved molecular atlas of c. elegans embryogenesis at single-cell resolution. *Science* **365**, eaax1971 (2019).

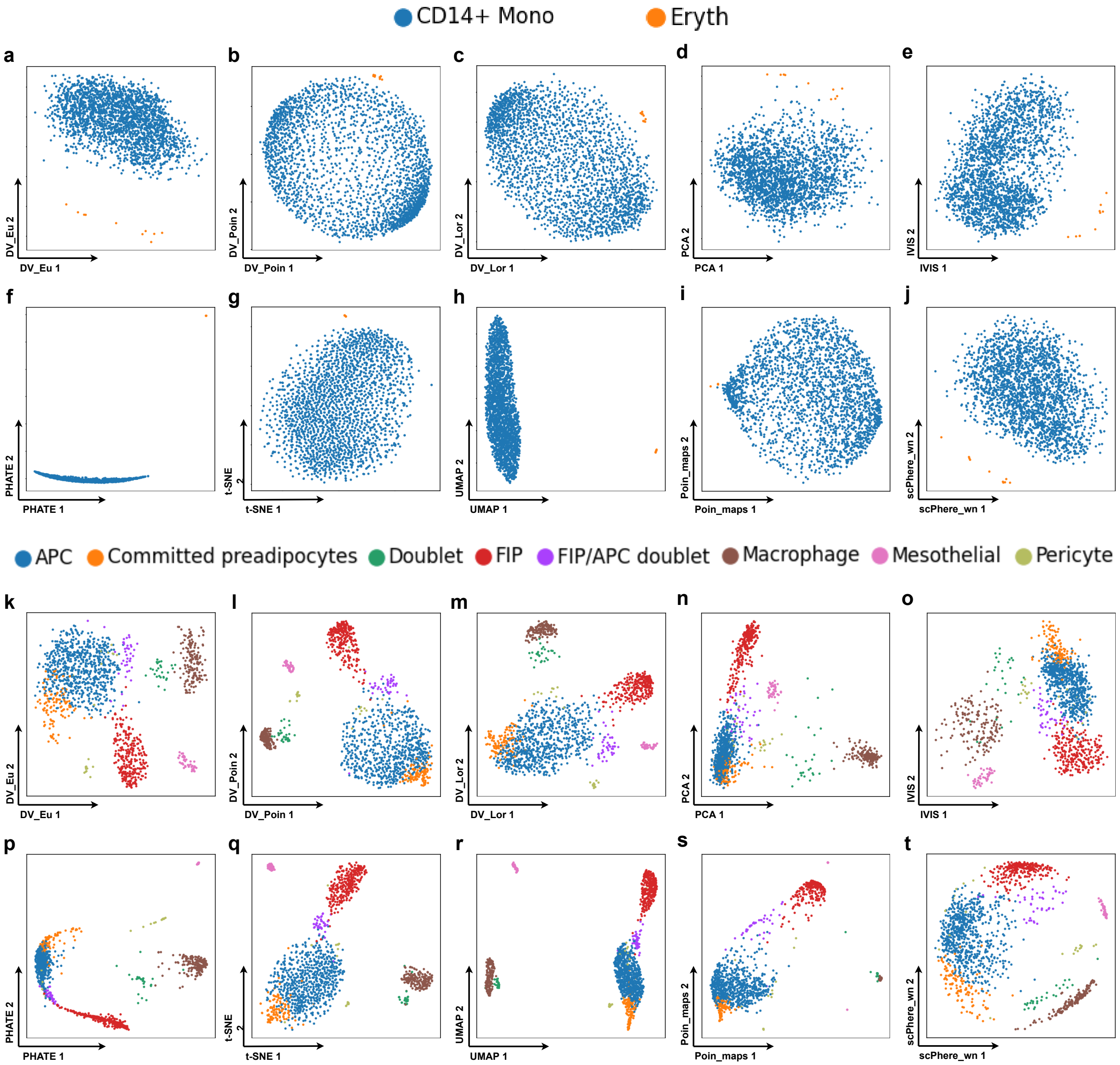

Supplementary Figure 1. DV visualizes small scRNA-seq data. a-t DV learns latent representations that provide excellent visualization in small datasets. DV_Eu learns representations in the Euclidean space (**a, k**). DV_Poin learns representations in the hyperbolic space with Poincaré model (**b, l**). DV_Lor learns representations in the hyperbolic space with Lorentz model (**c, m**). 2-dimensional PCA (**d, n**), IVIS (**e, o**), PHATE (**f, p**), t-SNE (**g, q**), UMAP (**h, r**), Poin_maps (**i, s**) and scPhere_wn (**j, t**) for CD14+ monocytes (**a-j**) and human lung cells (GSE130148) (**k-t**) with cells colored by cell types.

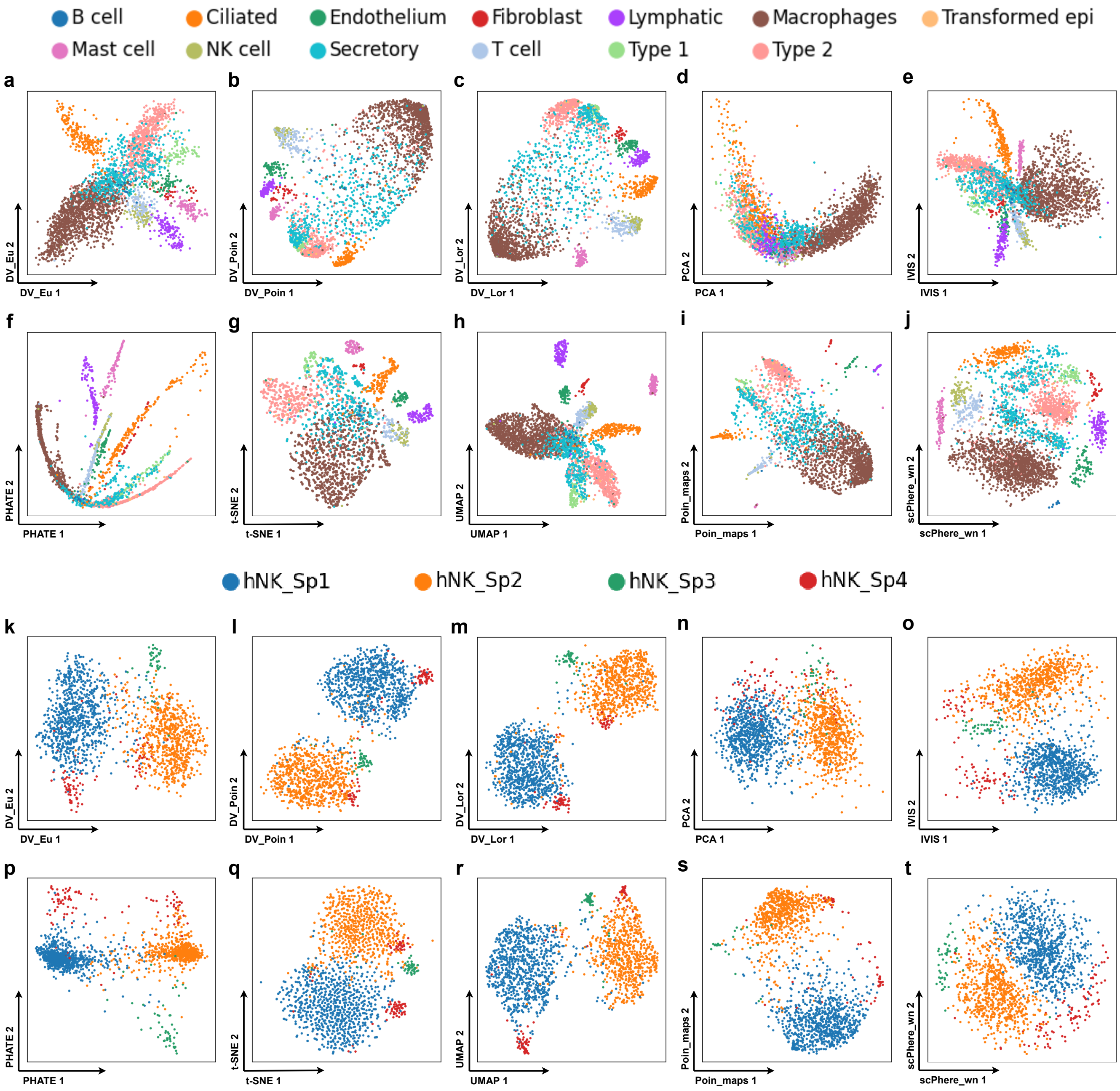

Supplementary Figure 2. **DV visualizes small scRNA-seq data.** **a-t** DV learns latent representations that provide excellent visualization in small datasets. DV_Eu learns representations in the Euclidean space (**a, k**). DV_Poin learns representations in the hyperbolic space with Poincaré model (**b, l**). DV_Lor learns representations in the hyperbolic space with Lorentz model (**c, m**). 2-dimensional PCA (**d, n**), IVIS (**e, o**), PHATE (**f, p**), t-SNE (**g, q**), UMAP (**h, r**), Poin_maps (**i, s**) and scPhere_wn (**j, t**) for mouse white adipose tissue stromal cells (GSE111588) (**a-j**) and human splenic nature killer cells (GSE119562) (**k-t**) with cells colored by cell types.

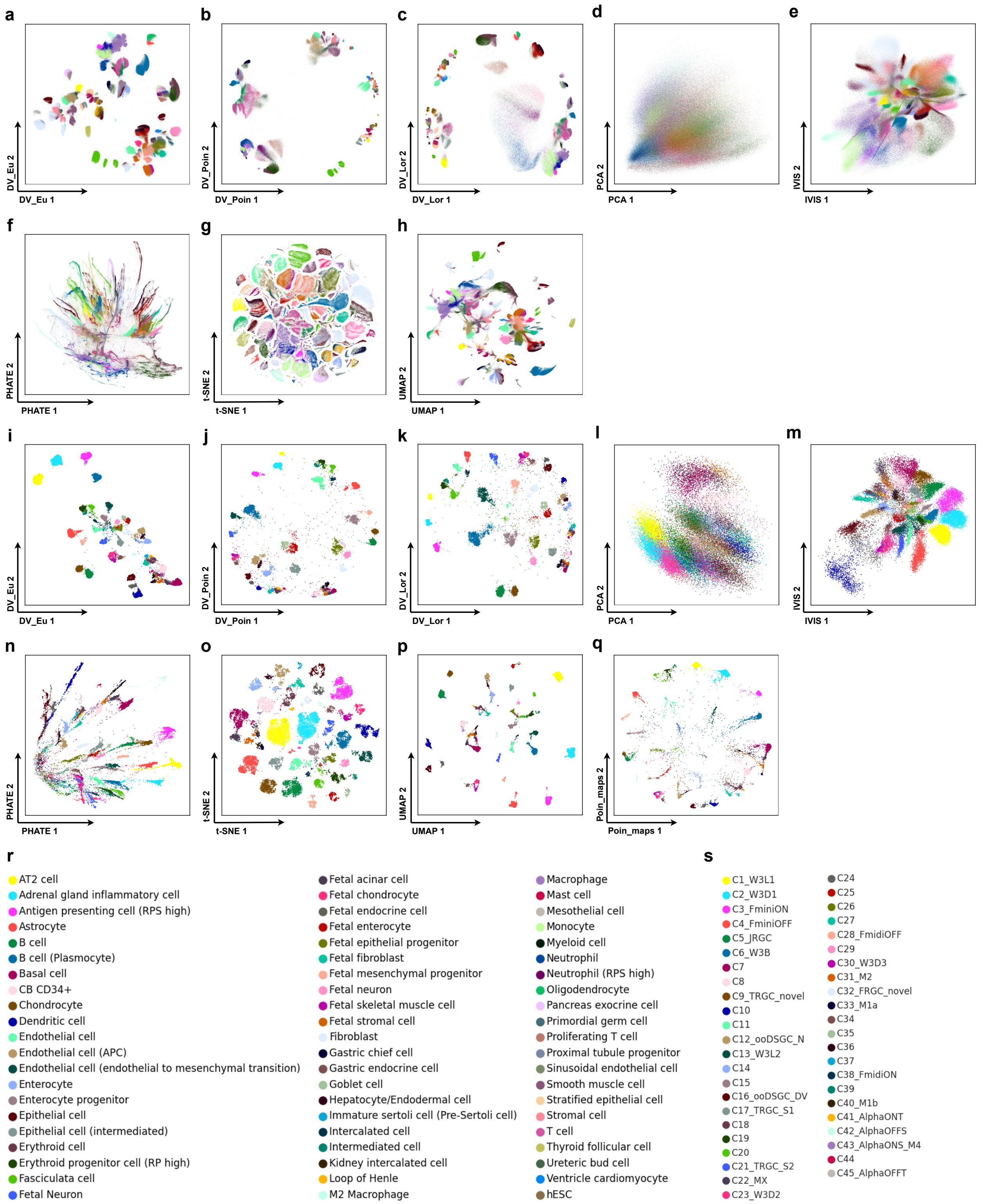

Supplementary Figure 3. DV preserves local and global structures in visualizing large *static* scRNA-seq data. a-q DV learns latent representations that provide excellent visualization of local and global structure, even in very large datasets. DV_Eu learns representations in the Euclidean space (**a, i**). DV_Poin learns representations in the hyperbolic space with Poincaré model (**b, j**). DV_Lor learns representations in the hyperbolic space with Lorentz model (**c, k**). 2-dimensional PCA (**d, l**), IVIS (**e, m**), PHATE (**f, n**), t-SNE (**g, o**), UMAP (**h, p**) and Poin_maps (**q**) for human cell landscape (HCL) (GSE134355) (**a-h**) and 45 mouse retinal ganglion cells (RGC) (GSE137400) (**i-q**) with cells colored by cell types.

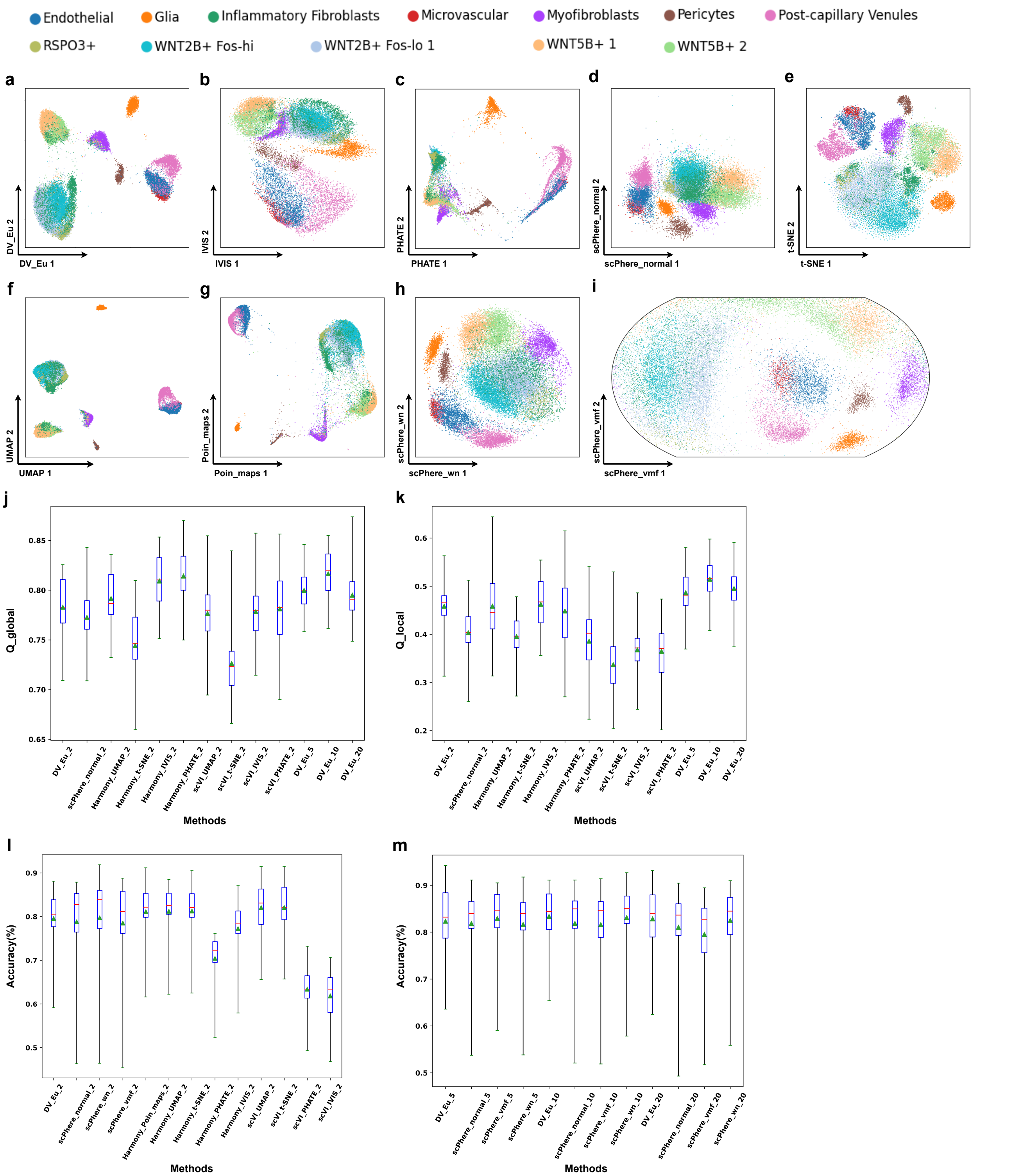

Supplementary Figure 4. DV addresses complex technical and biological batch for visualization and analysis in colon biopsies (SCP259) from healthy individuals and UC stromal patients. 2-dimensional DV_Eu (**a**), scPhere_normal (**d**), scPhere_wn (**h**) and scPhere_vmf (**i**) embedding accounting for the patient and disease status. 2-dimensional IVIS (**b**), PHATE (**c**), t-SNE (**e**), UMAP (**f**) and Poin_maps (**g**) embedding (batch-corrected by Harmony accounting for the patient status). Successful batch correction visualization as reflected by local (**j**) and global (**k**) geometric structure preservation performance (y axis), and *k*-NN classification accuracies (y axis, *k*=5) in 2-dimensional (**l**) and low-dimensional(**m**) embeddings. The geometric structure preservation is tested on the cells from one patient between input of model and visualization embeddings. The *k*-NN classification accuracy is tested on the cells from one patient after training on the cells from all other patients. Boxplots denote means, medians and interquartile ranges (IQRs). The whiskers of a boxplot are the lowest datum still within 15 IQR of the lower quartile and the highest datum still within 15 IQR of the upper quartile.

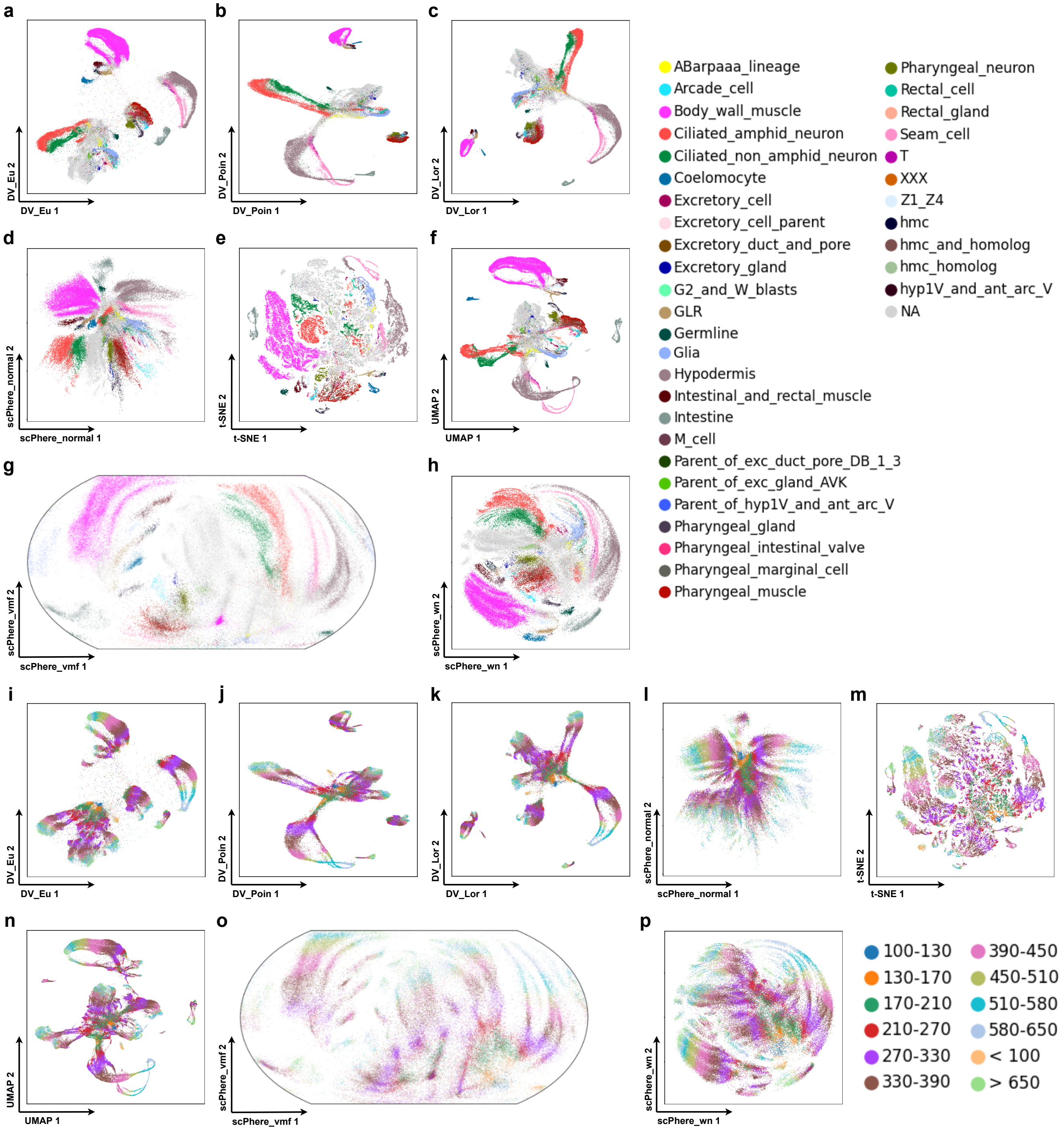

Supplementary Figure 5. DV shows developmental trajectories in visualizing large scRNA-seq differentiated data. DV_Eu (**a, i**) and scPhere_normal (**d, l**) learn representations in the Euclidean space. DV_Poin (**b, j**) learns representations in the hyperbolic space with Poincaré model. DV_Lor (**c, k**) and scPhere_wn (**h, p**) learn representations in the hyperbolic space with Lorentz model. scPhere_vmf (**g, o**) learned representations in the hyperspherical space. 2-dimensional t-SNE (**e, m**) and UMAP (**f, n**) for ELEGAN (GSE126954) with cells colored by cell types (**a-h**) and time phases (**i-p**).

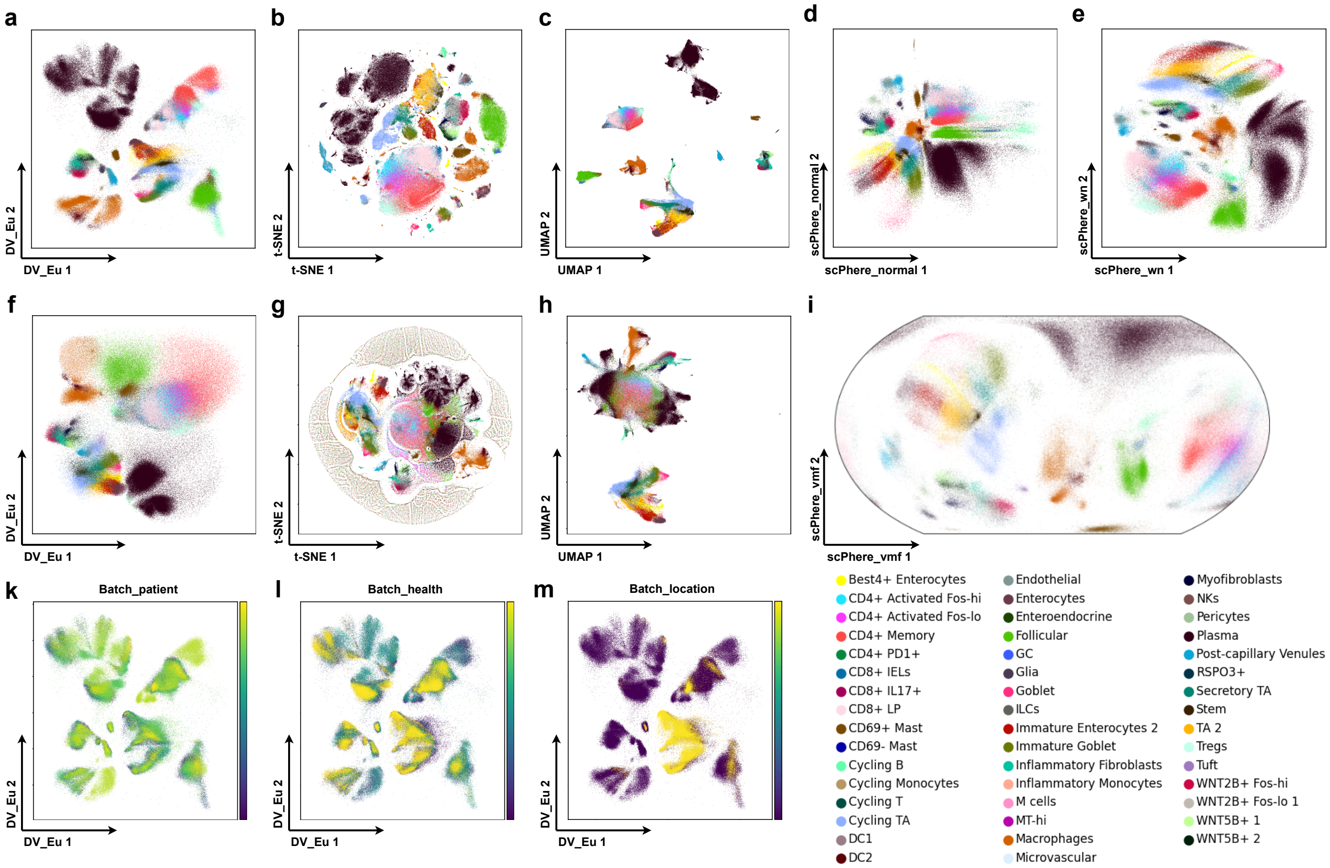

Supplementary Figure 6. DV addresses complex technical and biological batch for visualization and analysis in colon biopsies (SCP259) from healthy individuals, UC stromal, epithelial and immune patients. DV_Eu (**a**), scPhere_normal (**d**), scPhere_wn (**e**) and scPhere_vmf (**i**) embedding accounting for the patient, location, and disease status. 2-dimensional t-SNE (**b**) and UMAP (**c**) embedding (batch-corrected by Harmony accounting for the patient status). 2-dimensional DV_Eu (**f**) (remove PCA preprocessing), t-SNE (**g**) and UMAP (**h**) embedding (batch-corrected by Harmony accounting for the patient status when removing the PCA preprocessing). DV_Eu embedding colored by batch_patient (**k**), batch_health (**l**) and batch_location (**m**) label.

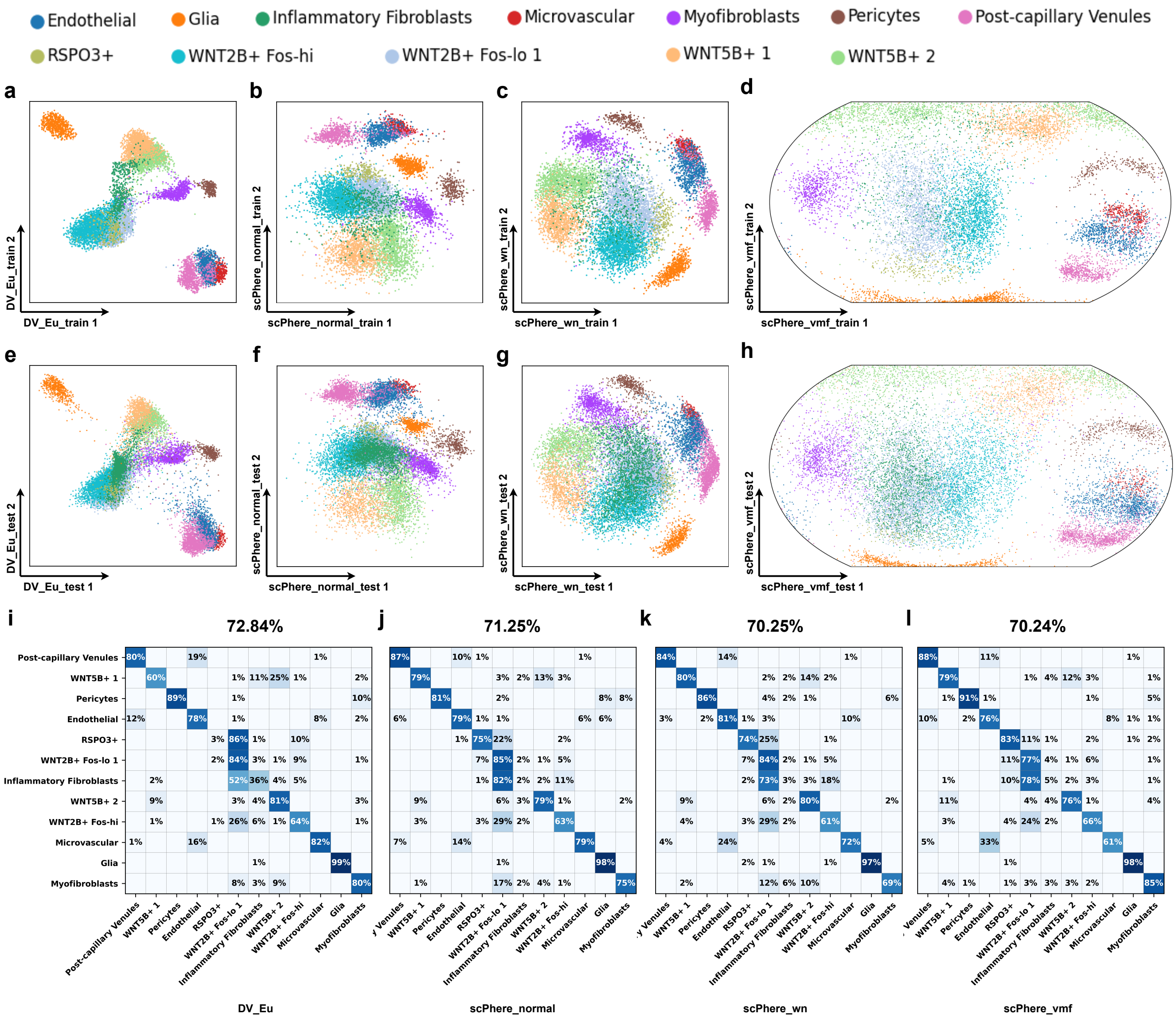

Supplementary Figure 7. Using complex batch vectors to pinpoint cell types impacted by biological factors and to generate a batch-invariant reference atlas. Batch-invariant DV trained on 18 patients (**a-d**) and test on 12 patients (**e-h**). DV_Eu learned representations in the Euclidean space (**a, e**). 2-dimensional scPhere_normal (**b, f**), scPhere_wn (**c, g**) and scPhere_vmf (**d, h**). Confusion matrices (**i-l**) of the overlap in cells (row-centered and scaled Z-score, color bar) between "true" cell types from the original study (rows) and cell assignment by k-NN classifications (k=5) from batch-invariant DV trained only on training set for stromal cells (SCP259).

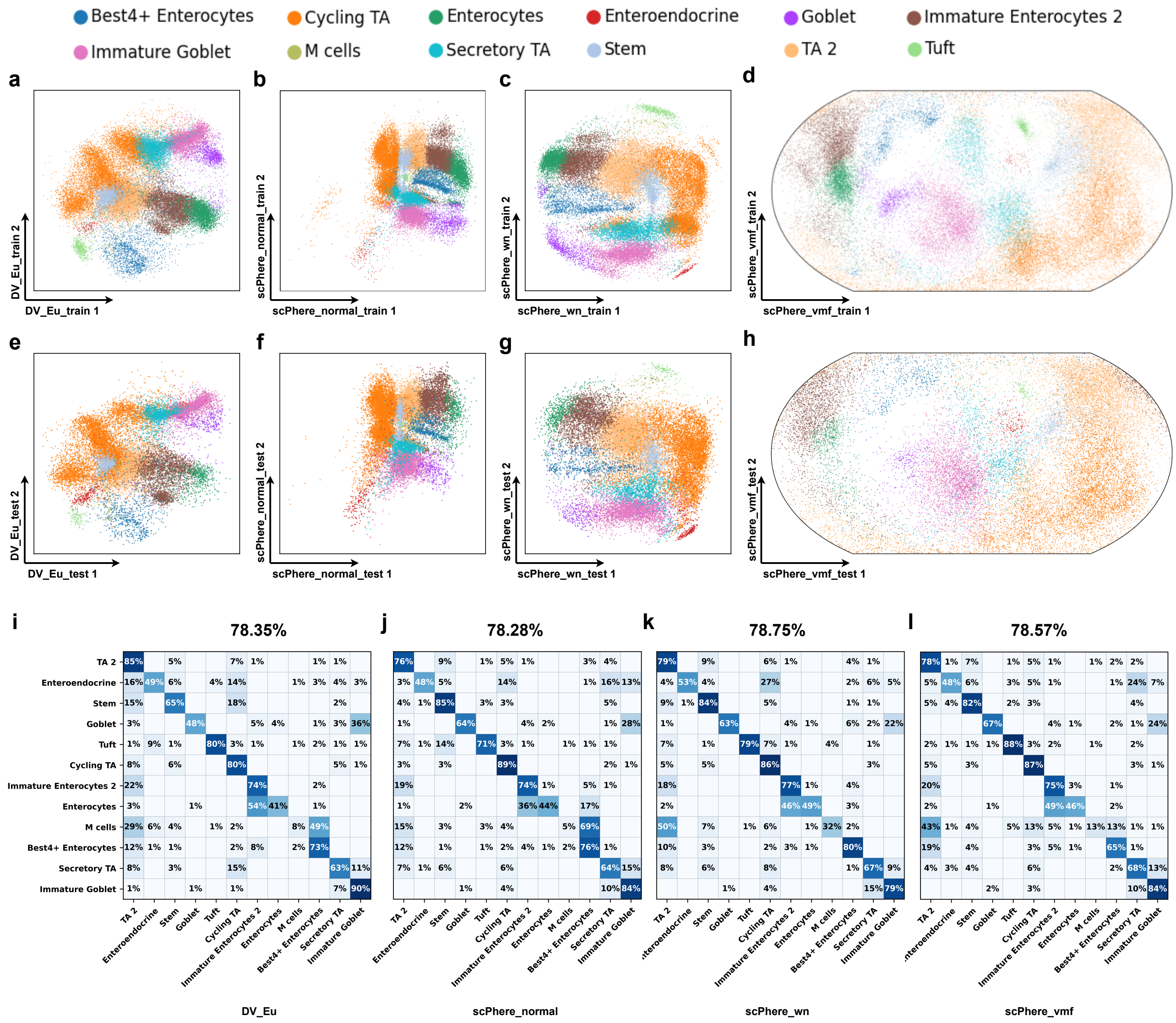

Supplementary Figure 8. Using complex batch vectors to pinpoint cell types impacted by biological factors and to generate a batch-invariant reference atlas. Batch-invariant DV trained on 18 patients (**a-d**) and test on 12 patients (**e-h**). DV_Eu learned representations in the Euclidean space (**a, e**). 2-dimensional scPhere_normal (**b, f**), scPhere_wn (**c, g**) and scPhere_vmf (**d, h**). Confusion matrices (**i-l**) of the overlap in cells (row-centered and scaled Z-score, color bar) between "true" cell types from the original study (rows) and cell assignment by k-NN classifications (k=5) from batch-invariant DV trained only on training set for epithelial cells (SCP259).

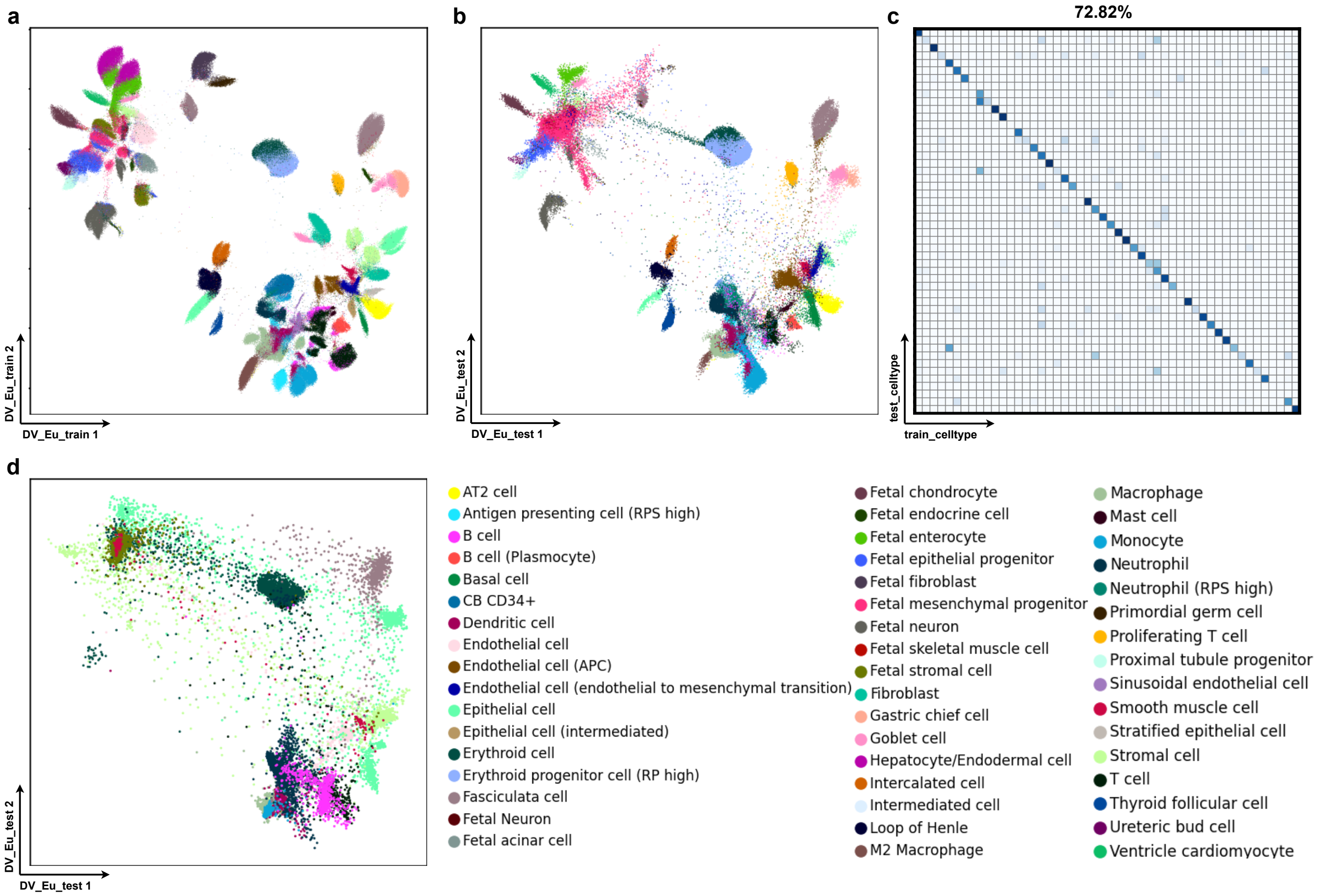

Supplementary Figure 9. DV generates a pretrained reference atlas and maps heterogeneous scRNA-seq data. The pre-trained DV trained on HCL cells (GSE134355) from 43 tissues (**a**), and test on HCL cells from 28 tissues (**b**) and MCA cells (**d**) test data. Confusion matrices (**c**) of the overlap in cells (row-centered and scaled *Z*-score, color bar) between “true” cell types from the original study (rows) and cell assignment by *k*-NN classifications (k=5) from batch-invariant DV trained only on training set for HCL cells.

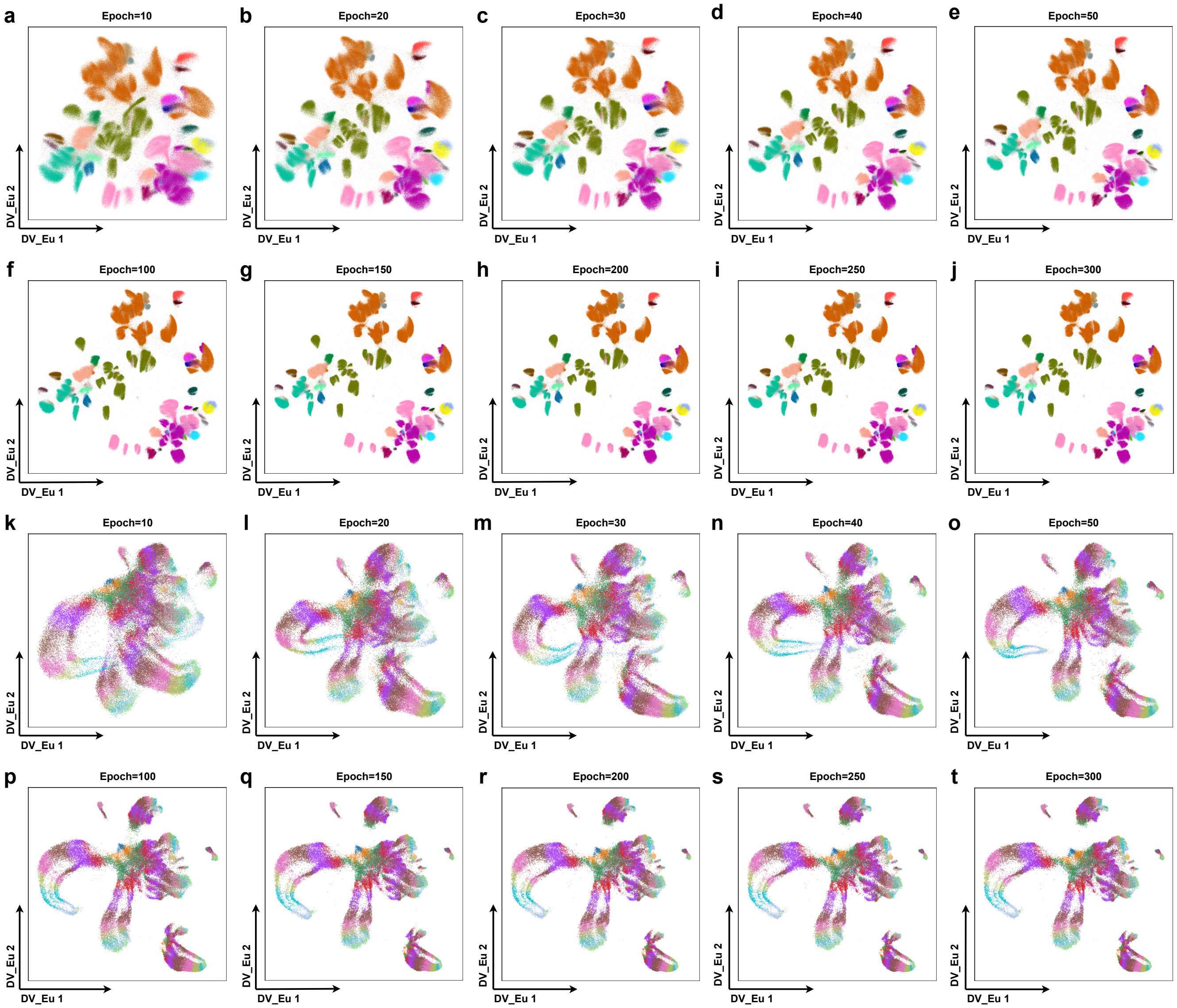

Supplementary Figure 10. DV converges rapidly on large *static* and *dynamic* scRNA-seq data without batch effect problem. DV visualization results with different epochs (10, 20, 30, 40, 50, 100, 150, 200, 250, 300) for human cell landscape (HCL) (GSE134355) (**a-j**) and C. elegans embryonic (ELEGAN) (GSE126954) (**k-t**).

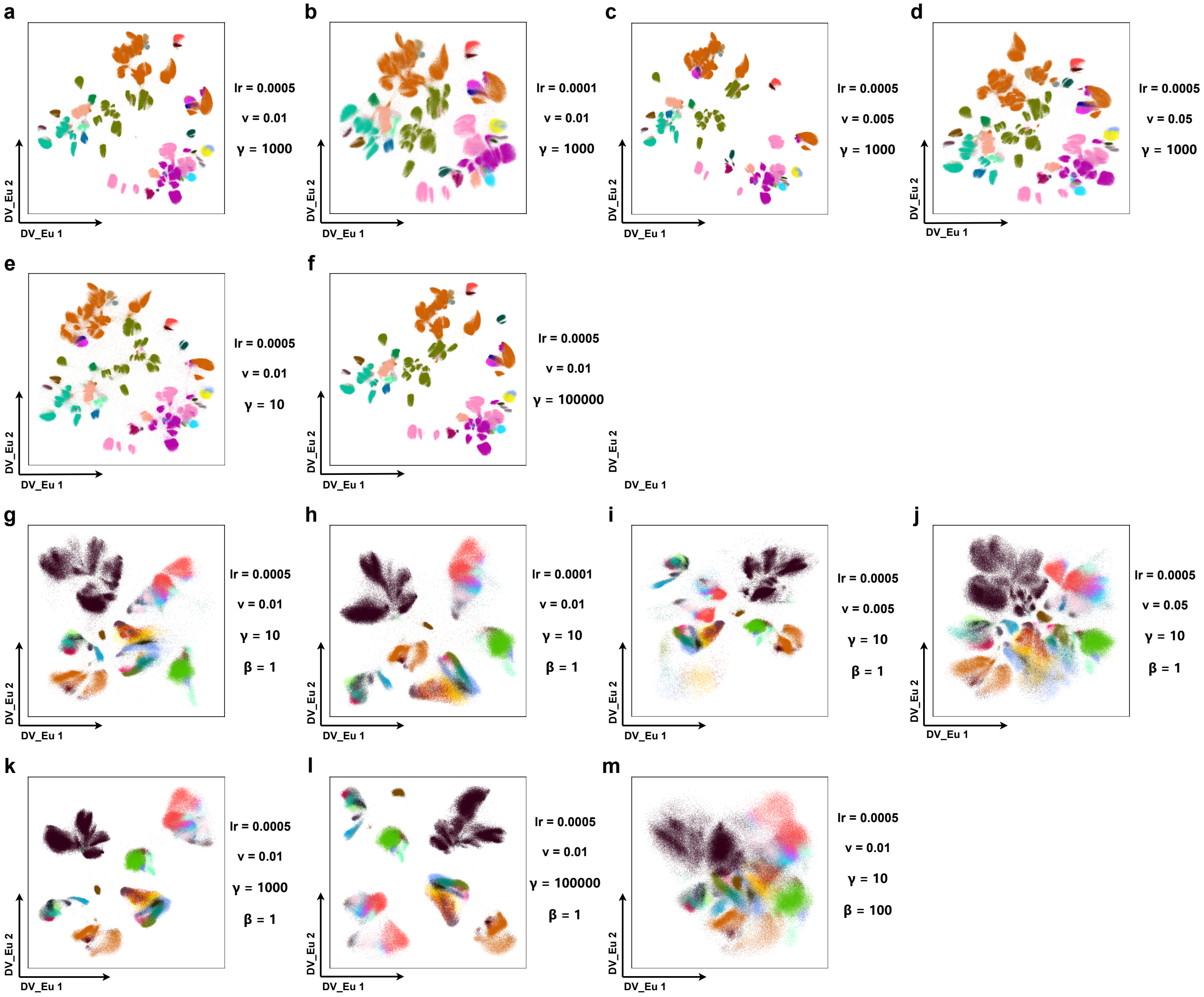

Supplementary Figure 11. DV_Eu is robust to hyperparameter on processing large *static* scRNA-seq data with/without batch effect problem. DV visualization results with different hyperparameters (learning rate lr, v, γ, β) for human cell landscape (HCL) (GSE134355) (**a-f**) and colon biopsies (SCP259) from healthy individuals, UC stromal, epithelial and immune patients (**g-m**).

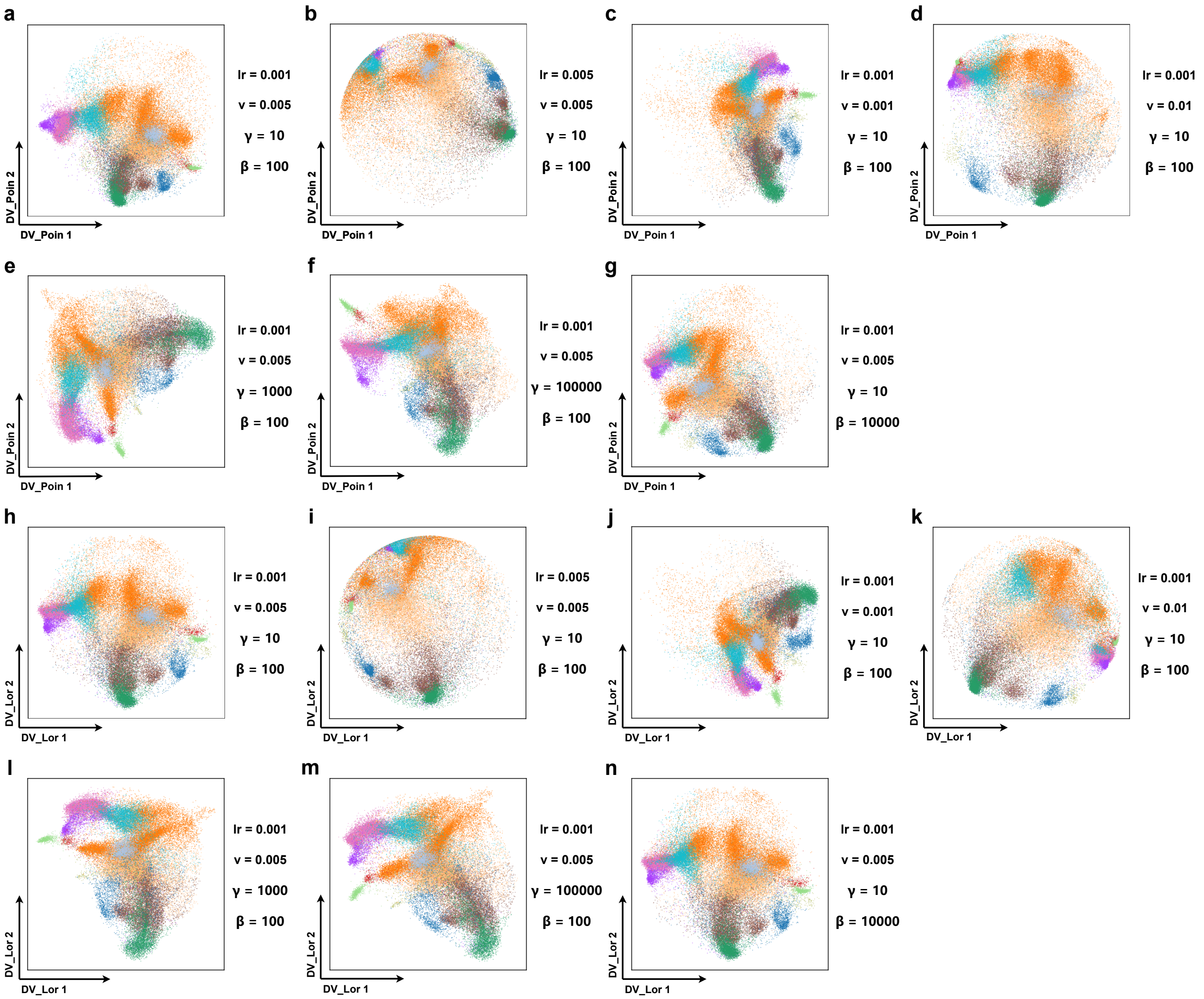

Supplementary Figure 12. DV_Poin and DV_Lor is robust to hyperparameter on processing *dynamic* scRNA-seq data with batch effect problem. DV_Poin (**a-g**) and DV_Lor (**g-n**) visualization results with different hyperparameters (learning rate lr, v, γ, β) for colon biopsies (SCP259) from healthy individuals and UC epithelial patients.

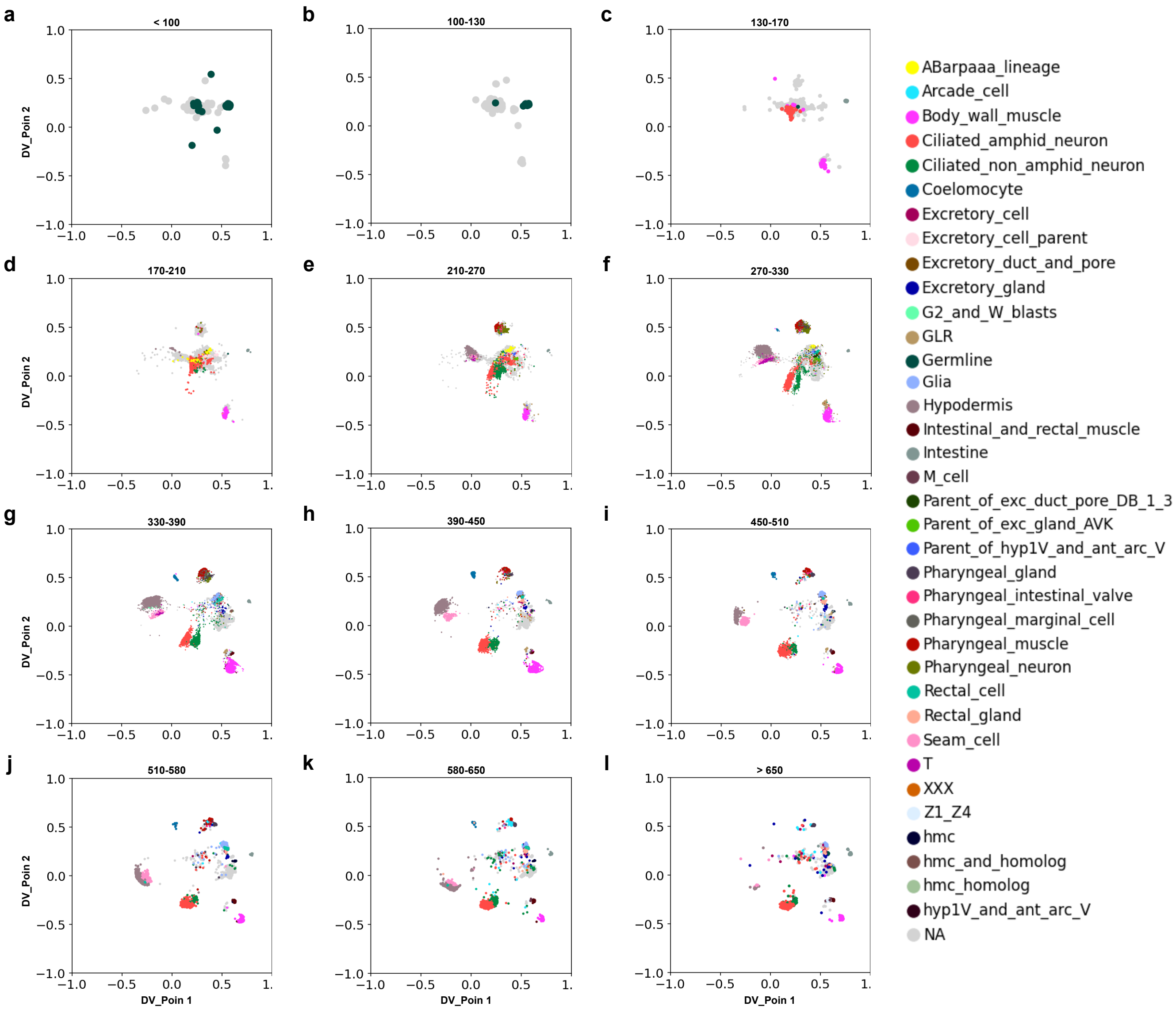

Supplementary Figure 13. Poincaré disk embedding of DV_Poin highlights the progression of C. elegans embryonic cells (GSE126954) in time without batch effect problem. Embedding of all C. elegans embryonic cells in a Poincaré disk, with each panel showing only the cells from one of 12 embryonic time bins. Cells are colored by annotated cell types.

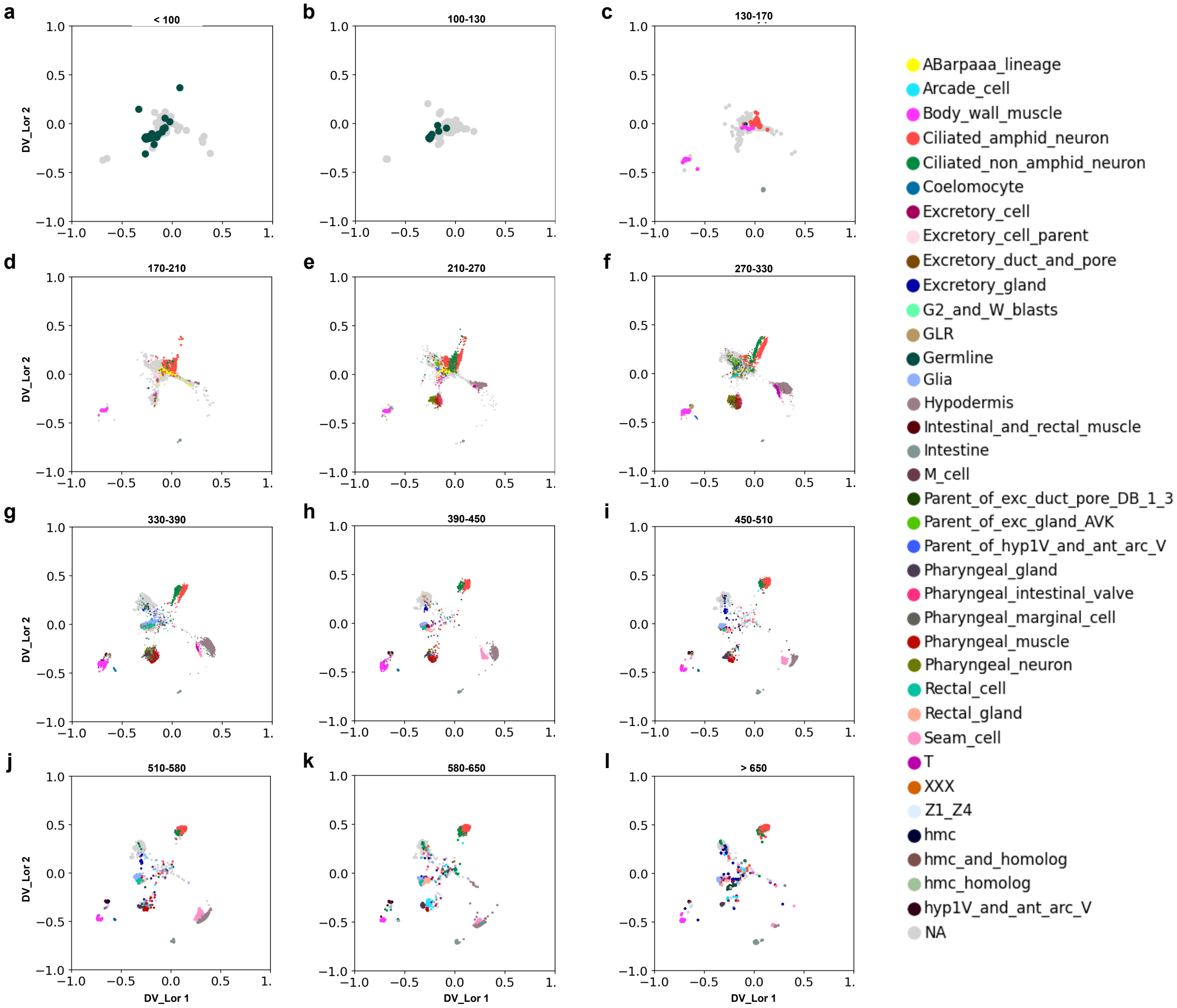

Supplementary Figure 14. Poincaré disk embedding of DV_Lor highlights the progression of C. elegans embryonic cells (GSE126954) in time with batch effect problem. Embedding of all C. elegans embryonic cells in a Poincaré disk, with each panel showing only the cells from one of 12 embryonic time bins. Cells are colored by annotated cell types.

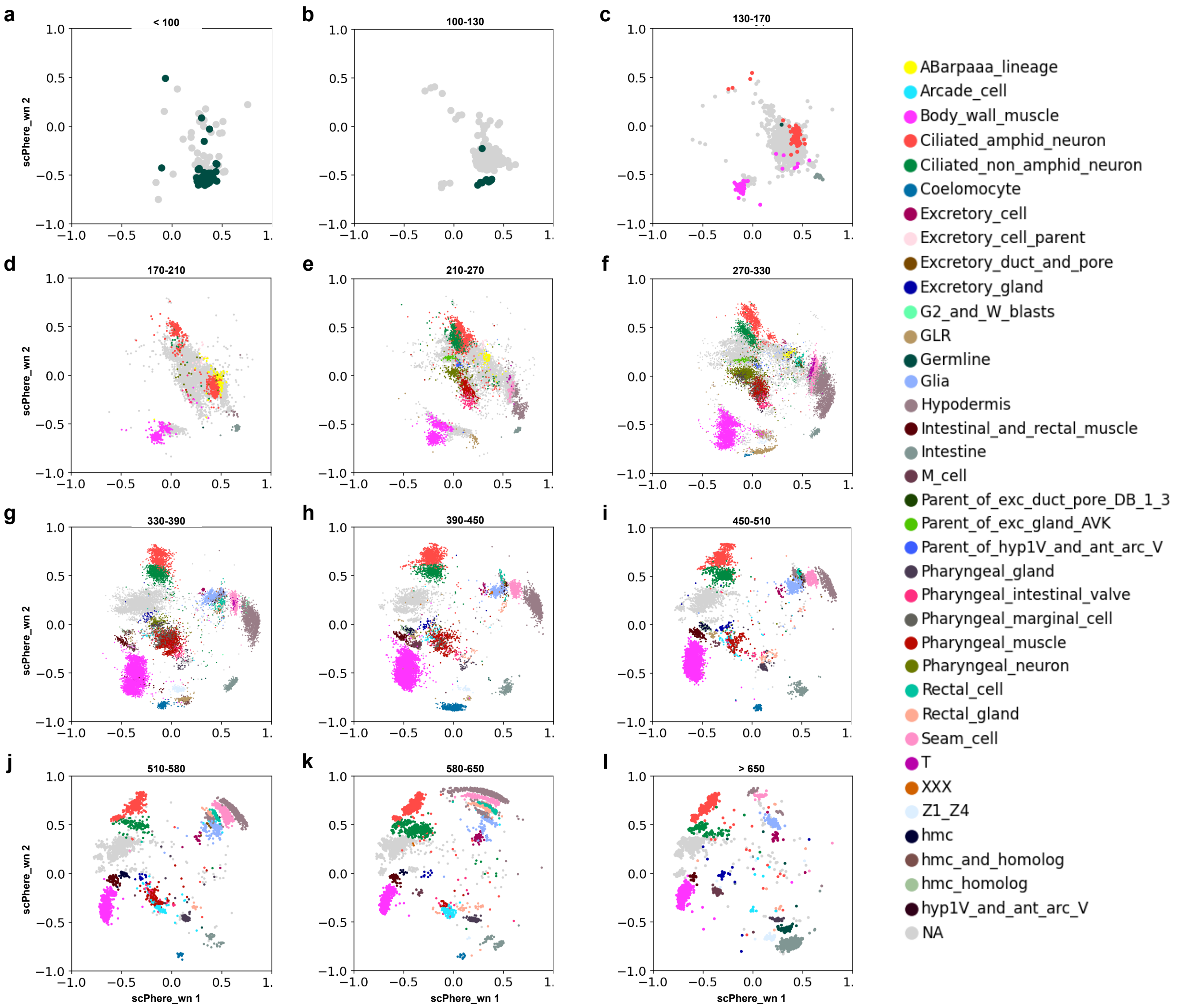

Supplementary Figure 15. Poincaré disk embedding of scPhere_wn highlights the progression of C. elegans embryonic cells (GSE126954) in time with batch effect problem. Embedding of all C. elegans embryonic cells in a Poincaré disk, with each panel showing only the cells from one of 12 embryonic time bins. Cells are colored by annotated cell types.

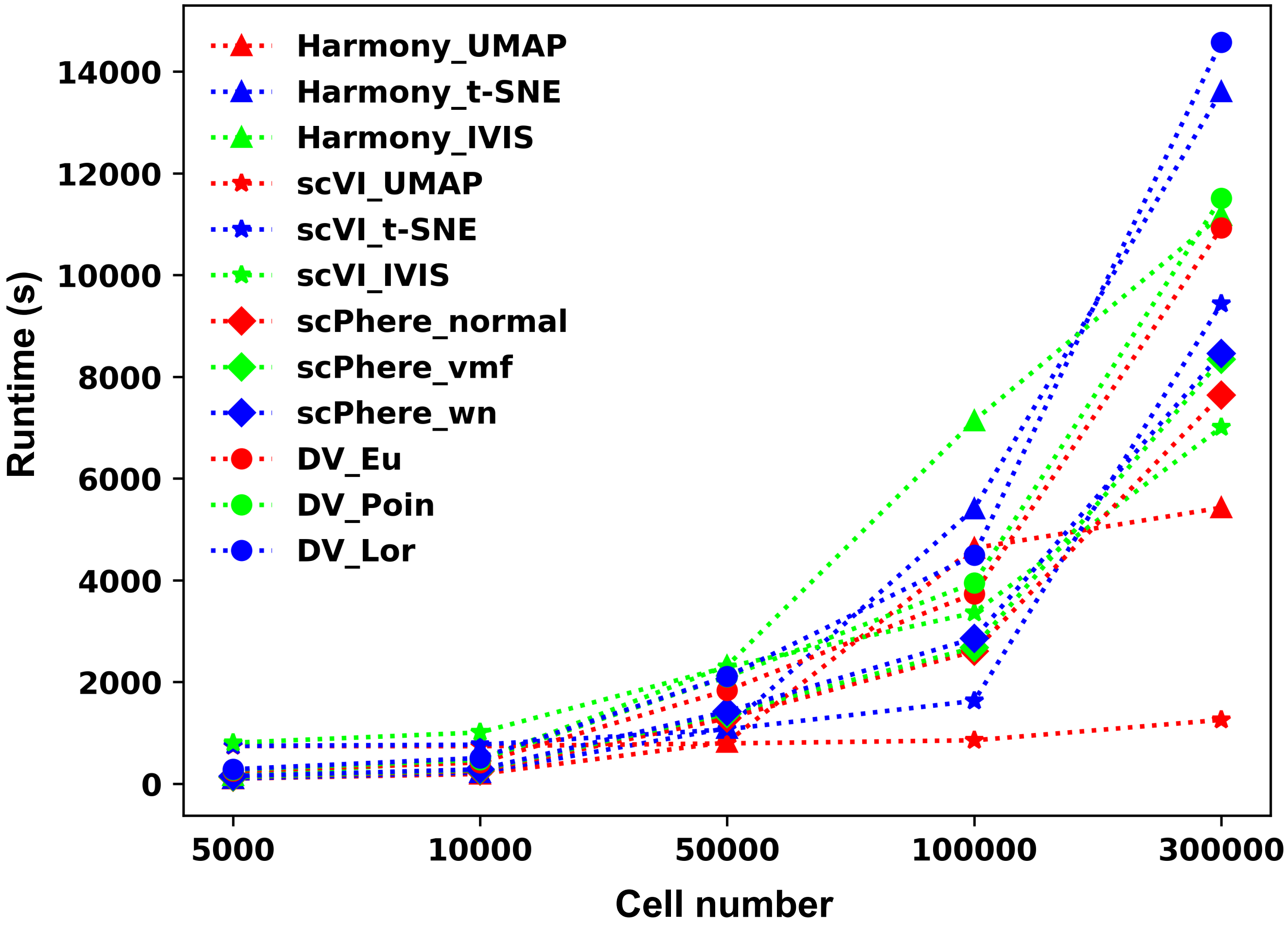

Supplementary Figure 16. Runtime of all methods when visualizing single-cell datasets (with batch effect problem) with different cell numbers. The cell number is set to 5000, 10000, 50000, 100000 and 300000.

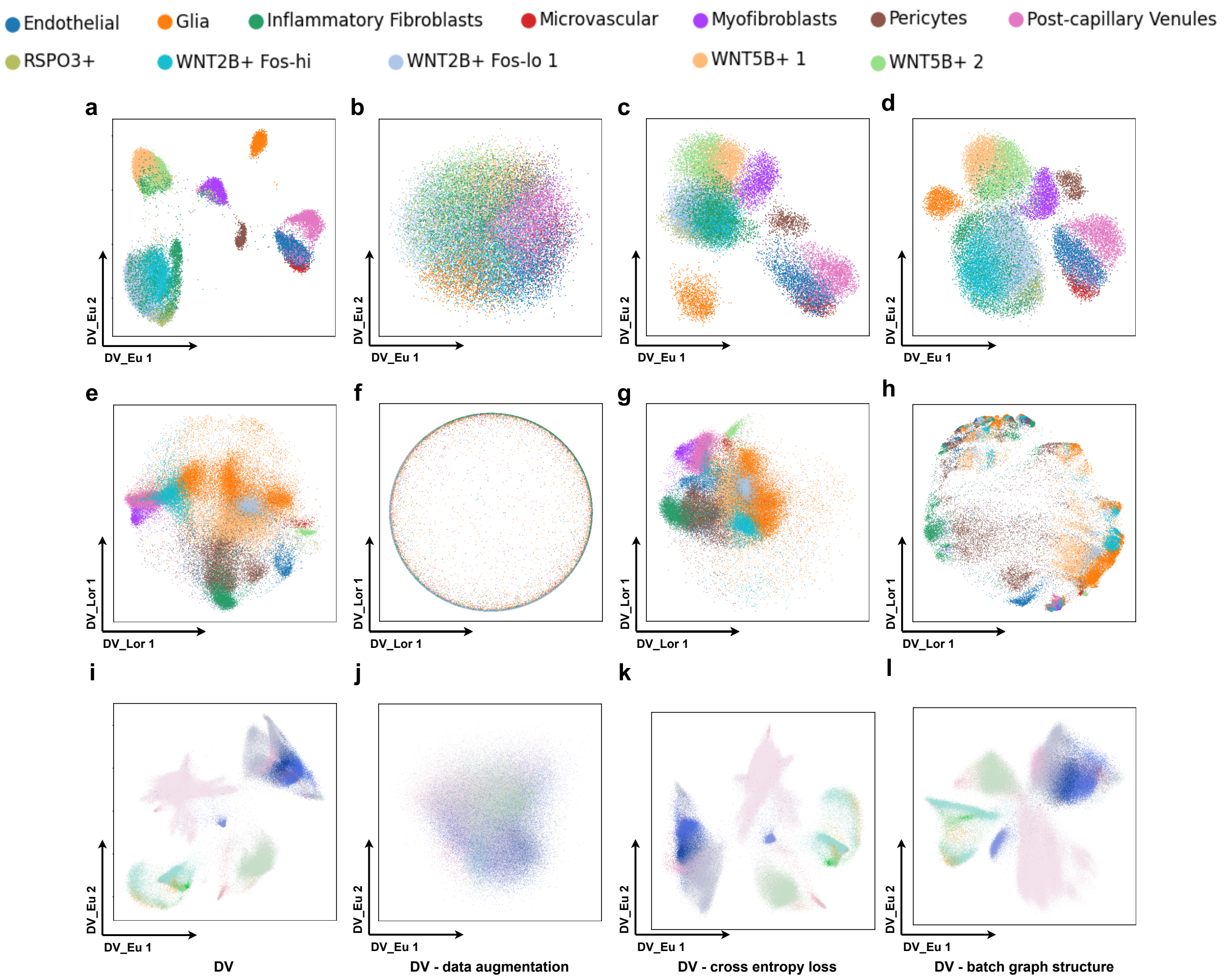

Supplementary Figure 17. Ablation study of DV when visualizing single cell datasets with batch effect problem. We mainly assess the importance of data augmentations (**b, f, j**), cross-entropy loss (**c, g, f**) and batch graph structure (**d, h, l**) in DV_Eu on the UC stromal (**a-d**) and immune datasets (**i-l**) and DV_Lor (**e-h**) on the UC epithelial dataset.
