# Supplementary material for: Structure-preserving visualization for single-cell RNA-Seq profiles using deep manifold transformation with batch-correction
Source: Commun Biol. 2023 Apr 4;6:369. doi: 10.1038/s42003-023-04662-z (PMC10073100; doi:10.1038/s42003-023-04662-z)
Supplement: Supplementary file 3 — Description of Additional Supplementary Files [file 42003_2023_4662_MOESM3_ESM.pdf]

Description of Additional Supplementary Files

File name: Supplementary Data 1

Description: The local and global geometric structure preservation performance of DV and baseline methods in RGC and HCL datasets

File name: Supplementary Data 2

Description: The local and global geometric structure preservation performance of DV and baseline methods in UC epithelial and immune cells from 30 patients with patient origin factor

File name: Supplementary Data 3

Description: The classification accuracy of DV and baseline methods in UC immune cells from 30 patients with patient origin factor

File name: Supplementary Data 4

Description: The confusion matrix of the overlap in cells between “true” cell types from the original study and cell assignments by k-NN classifications from batch-invariant DV and baseline methods trained only on training sets cells for UC immune cells.
