# Supplementary material for: Structure-preserving visualization for single-cell RNA-Seq profiles using deep manifold transformation with batch-correction
Source: Commun Biol. 2023 Apr 4;6:369. doi: 10.1038/s42003-023-04662-z (PMC10073100; doi:10.1038/s42003-023-04662-z)
Supplement: Supplementary file 8 — Reporting Summary [file 42003_2023_4662_MOESM8_ESM.pdf]

nature portfolio

Corresponding author(s): Stan Z. Li

Last updated by author(s): Jan 17, 2023

Reporting Summary

Nature Portfolio wishes to improve the reproducibility of the work that we publish. This form provides structure for consistency and transparency in reporting. For further information on Nature Portfolio policies, see our Editorial Policies and the Editorial Policy Checklist.

Statistics

For all statistical analyses, confirm that the following items are present in the figure legend, table legend, main text, or Methods section.

n/a	Confirmed	
☐	☒	The exact sample size (*n*) for each experimental group/condition, given as a discrete number and unit of measurement
☐	☒	A statement on whether measurements were taken from distinct samples or whether the same sample was measured repeatedly
☐	☒	The statistical test(s) used AND whether they are one- or two-sided<br>*Only common tests should be described solely by name; describe more complex techniques in the Methods section.*
☒	☐	A description of all covariates tested
☐	☒	A description of any assumptions or corrections, such as tests of normality and adjustment for multiple comparisons
☐	☒	A full description of the statistical parameters including central tendency (e.g. means) or other basic estimates (e.g. regression coefficient) AND variation (e.g. standard deviation) or associated estimates of uncertainty (e.g. confidence intervals)
☒	☐	For null hypothesis testing, the test statistic (e.g. *F*, *t*, *r*) with confidence intervals, effect sizes, degrees of freedom and *P* value noted<br>*Give P values as exact values whenever suitable.*
☒	☐	For Bayesian analysis, information on the choice of priors and Markov chain Monte Carlo settings
☒	☐	For hierarchical and complex designs, identification of the appropriate level for tests and full reporting of outcomes
☒	☐	Estimates of effect sizes (e.g. Cohen's *d*, Pearson's *r*), indicating how they were calculated

Our web collection on statistics for biologists contains articles on many of the points above.

Software and code

Policy information about availability of computer code

Data collection	We use publicly available datasets and preprocess it based on "scanpy" package.
Data analysis	We develop a new visualization method deep visualization (DV). The DV software package, implemented in Pyotrch, is available free from https://github.com/Westlake-AI/DV (we will remove the access permission after the manuscript is accepted).

For manuscripts utilizing custom algorithms or software that are central to the research but not yet described in published literature, software must be made available to editors and reviewers. We strongly encourage code deposition in a community repository (e.g. GitHub). See the Nature Portfolio guidelines for submitting code & software for further information.

Data

Policy information about availability of data

All manuscripts must include a data availability statement. This statement should provide the following information, where applicable:

- Accession codes, unique identifiers, or web links for publicly available datasets
- A description of any restrictions on data availability
- For clinical datasets or third party data, please ensure that the statement adheres to our policy

We use publicly available datasets in this study (GEO: GSE12695431, GSE11956234, GSE13014832, GSE11158833, GSE13740035, GSE13435536, GSE12695437; Single Cell Portal: SCP259, SCP551. To make the results presented in this study reproducible, all processed data are available in Single Cell Portal SCP1873 (we will remove the access permission after the manuscript is accepted).

Human research participants

Policy information about studies involving human research participants and Sex and Gender in Research.

Reporting on sex and gender	We use publicly available datasets in this study and no human research participants.
Population characteristics	We use publicly available datasets in this study and no human research participants.
Recruitment	We use publicly available datasets in this study and no human research participants.
Ethics oversight	We use publicly available datasets in this study and no human research participants.

Note that full information on the approval of the study protocol must also be provided in the manuscript.

Field-specific reporting

Please select the one below that is the best fit for your research. If you are not sure, read the appropriate sections before making your selection.

☒ Life sciences ☐ Behavioural & social sciences ☐ Ecological, evolutionary & environmental sciences

For a reference copy of the document with all sections, see nature.com/documents/nr-reporting-summary-flat.pdf

Life sciences study design

All studies must disclose on these points even when the disclosure is negative.

Sample size	We apply DV to multiple scRNA-seq datasets from human and mouse, spanning from small (thousands) to very large (hundreds of thousands) of cells from one or multiple tissues, and with a small (two) to very large (dozens) of expected cell types.
Data exclusions	We use publicly available datasets in this study and no data exclusions.
Replication	We freeze the experimental seed and choose best hyper-parameters. Therefore, all attempts at replication are successful.
Randomization	We use multiple publicly available scRNA-seq datasets in this study.
Blinding	We use publicly available datasets in this study.

Reporting for specific materials, systems and methods

We require information from authors about some types of materials, experimental systems and methods used in many studies. Here, indicate whether each material, system or method listed is relevant to your study. If you are not sure if a list item applies to your research, read the appropriate section before selecting a response.

Materials & experimental systems

n/a	Involved in the study	
☒	☐	Antibodies
☒	☐	Eukaryotic cell lines
☒	☐	Palaeontology and archaeology
☒	☐	Animals and other organisms
☒	☐	Clinical data
☒	☐	Dual use research of concern

Methods

n/a	Involved in the study	
☒	☐	ChIP-seq
☒	☐	Flow cytometry
☒	☐	MRI-based neuroimaging
